# OP31 Incorporating Biological Drugs For Rheumatic Diseases In The Brazilian Unified Health System: Analyzing The Budget Impact Analyses

**DOI:** 10.1017/S0266462325100895

**Published:** 2025-12-29

**Authors:** Carla Gottgtroy, Rosangela Caetano

## Abstract

**Introduction:**

The advent of immunobiological drugs, with special storage, transport, administration, and care requirements, adds to budget impact analysis (BIA) challenges. We analyzed BIA studies used in the recommendations of the Brazilian National Committee for Health Technology Incorporation (CONITEC) related to the incorporation of immunobiological drugs for treating immune-mediated rheumatic diseases (IMRD) between 2012 and 2023.

**Methods:**

An analysis of the BIA records related to immunobiologicals for the treatment of IMRD was carried out and nine CONITEC reports were reviewed. All reports were assessed regarding the compliance of the national BIA guidelines (NBIAG), published by the Brazilian Ministry of Health in 2012. Additionally, five semi-structured interviews were conducted with different key players, academic experts in the field, to discuss specific aspects related to the challenges of BIA of these drugs in the Brazilian scenario.

**Results:**

One BIA was dated before the NBIAG and was excluded from the joint analysis. Within the reports, the economic adjustments had the highest degree of adequacy (100%), while the costs of introducing a new medicine were not included in any of the reports. Nine categories were identified through content analysis, including the insufficiency of the BIA carried out for the complexity of the scenario being analyzed and issues related to biosimilar drugs. All the interviewees felt that the BIA carried out were not done from the perspective of the Unified Health System (SUS) as a tripartite management and financing model.

**Conclusions:**

This research pointed to the need for methodological improvements in BIA and strict compliance with the methodology currently recommended, at the risk of high uncertainty in the proposed models. The BIA should also consider the complexity of the SUS, its financing and tripartite management, the judicialization of health, and the Brazilian reindustrialization policies under development.

